# Detecting rumor outbreaks in online social networks

**DOI:** 10.1007/s13278-023-01092-x

**Published:** 2023-06-01

**Authors:** Damian Frąszczak

**Affiliations:** Military University of Technology, Warsaw, Poland

**Keywords:** Online social network, Rumor outbreaks, Rumor detection, Information propagation

## Abstract

Social media platforms are broadly used to exchange information by milliards of people worldwide. Each day people share a lot of their updates and opinions on various types of topics. Moreover, politicians also use it to share their postulates and programs, shops to advertise their products, etc. Social media are so popular nowadays because of critical factors, including quick and accessible Internet communication, always available. These conditions make it easy to spread information from one user to another in close neighborhoods and around the whole social network located on the given platform. Unfortunately, it has recently been increasingly used for malicious purposes, e.g., rumor propagation. In most cases, the process starts from multiple nodes (users). There are numerous papers about detecting the real source with only one initiator. There is a lack of solutions dedicated to problems with multiple sources. Most solutions that meet those criteria need an accurate number of origins to detect them correctly, which is impossible to obtain in real-life usage. This paper analyzes the methods to detect rumor outbreaks in online social networks that can be used as an initial guess for the number of real propagation initiators.

## Introduction and research motivation

Nowadays, social media platforms displace traditional ways of communication and information exchange. This trend continues due to critical factors that online social media provide: no cost, easy access, and is always available. Moreover, they are in standby mode by default, bombing users with real-time notifications about online neighborhood updates. It makes users feel on time with all the information without the necessity for searching for them as all the updates are given to them immediately. In one moment, people are bombed with news, pictures, videos, etc. They cannot correctly assess the content and verify if it is true or false. They make this decision based on their subjective feeling about processed information (Pennycook & Rand 2021). Having mentioned properties of social media platforms create an excellent opportunity to share information containing malicious content. Recently, it has been observed that these incidents are increasing and can affect different aspects of life, i.e., impacting the election results or financial and mental situations (Higdon 2020; *Market chaos after fake Obama explosion tweet—ABC News (Australian Broadcasting Corporation)*, b.d.). Identifying the source of information is crucial as it can reduce disinformation and consequently avoid more severe problems (Frąszczak 2021a, 2022; Khan et al. 2021).

In most cases, fake news or rumors on social media platforms are initiated by groups of users located in separate neighborhoods. Many of those users are not real, just computer bots propagating appropriate messages easily attached or detached from the network. They are placed in different parts of the network as the organization aims to make immense desolation among the users and wants to cover the network with rumor content as soon as possible. It is reported that people believe in information and share it faster when it can be confirmed from multiple sources (Higdon 2020; Khan et al. 2021). Furthermore, it is harder to classify given content as fake when it is spread in numerous groups simultaneously than in one. Recently, it can be observed there have been developed multiple methods for identifying rumor sources in networks (Frąszczak 2021a; Jiang et al. 2017; Shelke & Attar 2019). Most of them are usable for single-source detection problems. Only some of them can be used for multi-source tasks. Unfortunately, most of them need a valid number of sources, which is impossible in real-life cases (Frąszczak 2021a; Shelke & Attar 2019). This paper investigates the problem by analyzing the available community detection methods and evaluating their accuracy. Furthermore, it also validates the accuracy of the well-known source detection problems over identifies outbreaks.

This paper is divided into six main parts. The first one introduces online social networks and their mathematical background. Furthermore, it also contains some essential information regarding rumor propagation and source detection. The second analyzes the techniques used for multi-source detection problems and their approach to finding rumor outbreaks. The third one introduces the simulation environment used to carry out the analysis of current methods. The fourth contains the simulation conditions, and the fifth describes the performed examinations and presents the obtained results in the simulation environment. The last one concludes the paper, summarizes the problems, and indicates possible future development directions in that area.

## Online social networks, rumor propagation, and source detection

Social media platforms are broadly used to exchange information by milliards of people worldwide. Each day people share a lot of their updates and opinions on various types of topics. Their popularity is due to critical factors, including quick and accessible communication via the Internet, availability, or free. The number of active users is still growing. For Facebook, this number in the five years increased twice, giving in 2020 about 2740 mln active users using that platform (Digital News Report, 2016, b.d.). These conditions make it easy to spread information from one user to another in close neighborhoods and around the whole social network on the given platform (Frąszczak 2021b; Guille et al. 2013; Mei Li et al. 2017). Unfortunately, it is reported that it has been recently increasingly used for malicious purposes, e.g., rumor propagation. As mentioned vicious incidents can impact different aspects of people’s lives, including election results, stock changes, or people’s lives (Higdon 2020). Identifying the source of malicious information is crucial as it can reduce disinformation and avoid more severe problems.

Online social networks are represented with the graph theory. Its structure is defined by the graph $G = (V,E)$, where $V$ represents a countably infinite set of nodes (users) and $E$ is a set of edges (relations between them) connected via an adjacency matrix. In most cases, the particular cells of this matrix contain “1” as a value for connected nodes “0” is used otherwise. The edges can represent the one-directional relationship between nodes representing an independent relationship, i.e., Twitter following the relation between two users (Raj et al. 2018). This type of association is called “directed.” In contrast, the two-directional relationship between nodes is called “undirected” and is used to model mutual relationships, i.e., Facebook friendship (Frąszczak 2021a).

Malicious information in online social networks can be initiated from a single or set of nodes, the rumor sources $v \subseteq G$. These nodes are called active or susceptible (Frąszczak 2021b; Mei Li et al. 2017; Rossetti et al. 2019; Tarapata & Kasprzyk 2010) and actively participate in a rumor-spreading process. It is achieved by passing information to its neighbors and encouraging them to participate in this action. Each user (node) that has started giving information further in a network becomes an active node, moving forward an information propagation process. An infection graph is created as time passes and more nodes become infected.$G_{I}$ is a subgraph of $G$ and consists of infected nodes $V_{I}$ which have taken part in information propagation via edges $E_{I}$[4], [7], [8]. The task of source detection methods is to find original rumor sources based on a given infected graph $G_{I}$(Frąszczak 2021a; Jiang et al. 2017; Shelke & Attar 2019). For problems dedicated to single sources, the whole infection graph is analyzed. For the multi-source ones, the number of sources is provided as input parameters or is estimated with community detection methods able to identify the number of them based on some metrics. Then the infection graph is either divided into smaller areas where each contains one source or is analyzed as one part, and estimated sources with the most considerable value are considered real ones (Shelke & Attar 2019; Zang et al. 2015). In this paper, the first approach is analyzed.

Multiple approaches exist to detect rumor sources and identify outbreaks based on the given infection graph. The most popular in recent research are maximum likelihood (ML) and maximum posteriori (MAP) estimations (Frąszczak 2021a; Shelke & Attar 2019). This paper analyzes the accuracy of those methods based on the identified outbreaks. One of the most straightforward and most used ML estimations to detect rumor sources is centrality measures broadly used to assess nodes in a network based on its structure (Ali et al. 2020; Das et al. 2018; Das & Kumar Sinha 2018). There is an assumption that information should be initiated from the most valuable nodes to reach as big as possible network coverage in the shortest time. Therefore new centrality metrics are still discovered and used for various problems (Chebotarev & Gubanov 2020). The most famous six in rumor source detection are used for this paper and introduced in Table 1. The usefulness of well-known single-source detection methods such as NetSleuth (Prakash et al. 2012), Rumor Center (Dong et al. 2013; Shah & Zaman 2010, 2011), and Jordan Center (Zhu & Ying 2013) applied with the divide and conquered approach is also analyzed.

**Table 1:** Most famous centrality metrics used in rumor source detection research

Centrality measure/time complexity	Formula	Analysis	Application area
Degree centrality $O(m)$	$C_{D} (x) = d_{x}$	Counts edges incident to a node	Determining popular users
Closeness centrality $O(n^{3} )$	$C_{C} (x) = \frac{1}{{\sum\nolimits_{y \in N} {d(x,y)} }}$ where $d(x,y)$ is the geodesic distance between the nodes *x* and *y*	Distance from one node to others	Determining a location that can spread information fast
Eccentricity/Jordan/Radius centrality $O(mn)$	$C_{EC} (x)\frac{1}{{\max_{y \in N} d(x,y)}}$	Maximum distance between nodes	Determining a location that can spread information fast
Betweenness centrality $O(n^{3} )$	$C_{B} (x) = \sum\limits_{y \ne z \in N} {\frac{{\sigma_{st} (x)}}{{\sigma_{st} }}}$ $\sigma_{st}$ number of all shortest paths between *s* and *t* in the network $\sigma_{st} (x)$- number of all shortest paths between *s* and *t* in the network including x	Counts the number of the shortest paths passing through the node	Determining the node that controls the information among other nodes
Eigenvector centrality $O(n^{2} )$	$Ax = \lambda x,\lambda x_{i} = \sum\limits_{j}^{n} {a_{ij} x_{j} }$ $a_{ij}$ the cell in adjacency matrix A	Counts important links	Finding nodes connected with many high-scoring nodes. Determining the location of the emergency facility
Rumor centrality $O(n^{3} )$	$R(i,G) = \prod\limits_{u \in G} {\frac{N!}{{T_{u}^{v} }}}$ where $u$ is a node of $G$ and $T_{u}^{v}$ is the number of nodes in the subtree rooted at *u* with *v* as the source	Counts the number of possible propagation permutations from node	Determining rumor source

## Current solutions for multi-source detection problems

The main research interest for source detection problems is focused on single-source issues. Unfortunately, fake news propagation in real-world situations is initiated by multiple sources (Jiang et al. 2017; Nguyen et al. 2016; Zang et al. 2014). The multi-source detection problem is more complex than the single one because the node evaluation must be computed for all possible subsets of infected nodes. The complexity of generating all possible subsets of possible source nodes assumes that source nodes are equal to $$\left( {\begin{array}{*{20}c} {\left| {V_{I} } \right|} \\ m \\ \end{array} } \right)$$. When there are many infected nodes, $V_{I} = O(n)$ there would be $O(mn)$ possible source subsets. This fact makes the multi-source detection problem computationally hard for large networks, even for small values $m$. Most of the available solutions for multi-source detection techniques utilize the divide and conquer approach to divide multi-source detection problems into a single source and then evaluate well-known methods for single-source detection. This division is obtained via network partitioning or community detection algorithms (Luo et al. 2014; Zang et al. 2014). Moreover, most cases use network partitioning or community detection methods that require an expected number of partitions, making the solution not usable for real-life problems where it is not known. Some solutions utilize different approaches to introduced ones: ranking and approximation based. More details about them can be found in (Choi et al. 2020a, b; Shelke & Attar 2019; Zhang et al. 2017).

The main task for both community detection and network partitioning methods is to find groups (clusters/communities) in the network, so each node belongs to one group. Desirable groups are densely connected to the nodes in the same group and sparsely connected to nodes in others. Sociology researchers noticed that individuals in the same community share similarities, such as gender, age, common interests, professional activity [32]. Therefore, the main aim of those methods is to detect groups of nodes that share similar properties and differ from other nodes concerning certain criteria. Those criteria are different for both approaches, as they emerge from different origins. Network partitioning techniques are based on graph theory, whereas community detection where developed based on sociology. Mathematically, network partitioning and community detection methods aim to divide $G$ into $q$ disjoint sub-graphs $C_{i} = (V_{i} ,E_{i} )$, in which $\forall i \ne j:C_{i} \cap C_{j} = \emptyset$ and $\bigcup\limits_{i = 1}^{k} {C_{i} = V}$. The evaluated communities are then estimated with quality functions. All evaluation metrics used to assess the obtained communities are introduced in Table 2. Those problems are NP-complete problems (Fortunato 2010) as there are an exponential number of various alternative partitions. Moreover, not all community detection methods can be used in all cases, as some are only dedicated to undirected and unweighted structures, while having such a complex input network structure is not recommended to abandon such detail, leading to inappropriate results.

**Table 2:** Community detection evaluation metrics

Name	Formula	Analysis
Modularity	$Q = \frac{1}{2m}\sum\limits_{i,j}^{{}} {\left[ {A_{i,j} - \frac{{k_{i} k_{j} }}{2m}} \right]} \,\delta \left( {C_{i} ,C_{j} } \right)$ where $m$- the total number of edges of the graph $\delta$- is the Kronecker delta function that yields one if nodes $i$ and $j$ are in the same community ($C_{i} = C_{j}$), zero otherwise	It compares the real network structure with a corresponding one where nodes are connected without preference for their neighbors (Newman & Girvan 2004)
Normalized mutual information—NMI	$NMI(A,B) = \frac{{ - 2\sum\nolimits_{i = 1}^{{C_{A} }} {\sum\nolimits_{j = 1}^{{C_{B} }} {M_{ij} \cdot \log \left( {\frac{{M_{ij} \cdot n}}{{M_{i} \cdot M_{j} }}} \right)} } }}{{\sum\nolimits_{i = 1}^{{C_{A} }} {M_{i} \cdot \log \left( {\frac{{M_{i} }}{n}} \right)} + \sum\nolimits_{j = 1}^{{C_{B} }} {M_{j} \cdot \log \left( {\frac{{M_{j} }}{n}} \right)} }}$ where $A,B$- partitions in graph $C_{A} ,C_{B}$- number of communities in partitions A and B, respectively $M_{ij} -$ element of the matrix $(M)\,C_{B} xC_{B}$, representing the number of nodes in the $i$th community of $A$ that appear in the *j*th community of $B$	The NMI is used to compare two sets of partitioning results. The value is high when the two results are similar. NMI is normally used when the ground truth of the network (the correctly partitioned set) is available (Danon et al. 2005)
Performance	$P(C) = \frac{{\left| {\left\{ {(i,i) \in E,C_{i} = C_{j} } \right\}} \right| + \left| {(i,j) \notin E,C_{i} \ne C_{j} \} } \right|}}{n(n - 1)/2}$	It counts the number of correctly “interpreted” pairs of vertices, i.e., two vertices belonging to the same community and connected by an edge, or two vertices belonging to different communities and not connected by an edge (Fortunato 2010)
Partition coverage	$C(C) = \frac{{m_{c} }}{m}$ where $m_{c}$- the number of edges in the community $C$	It is the ratio of the number of intra-community edges to the total number of edges in the graph (Fortunato 2010)

The method presented in (Luo & Tay 2012) utilized the Voronoi partitioning method to divide a network into multiple partitions, whereas the classical rumor center detection method is used to find a single source. In (Jiang et al. 2015) Capacity Constrained Network-Voronoi Diagram (CCNVD) (Yang et al. 2013) network partitioning method, together with a new metric of effective distance (Brockmann & Helbing 2013), was used for identifying multiple sources. Zang et al. (2014) used three different network division methods to detect numerous sources: leading eigenvector-based approach, edge betweenness, and mixed membership block model methods. The leading eigenvector-based method divides nodes into groups that satisfy two characteristics: sparse edges between different groups and abundant edges within the same group. This approach is also called a modularity-based one. Network partition utilizing edge betweenness divides nodes into groups by focusing on the boundaries of communities instead of their cores. The mixed membership block model divides nodes into groups because nodes infected by the same source are more likely to link, while nodes infected by different sources have less contact. An extension of the leading eigenvector-based method with modularity metrics was presented in (Zang et al. 2015). This paper also introduced a heuristic algorithm for estimating the number of sources utilizing the community detection algorithm.

Besides the methods used in the current research, some more community detection and network partitioning methods can be used to detect rumor outbreaks without the necessity of an expected number of groups. The following techniques can be modularity-based like the Louvain method (Blondel et al. 2008), label propagation (Cordasco & Gargano 2011), Clauset–Newman–Moore (Clauset et al. 2004) or Girvan-Newman (Girvan & Newman 2002), and much more (Frąszczak 2022). All used methods in the analysis are introduced further in the article.

Clauset–Newman–Moore’s (CNM) (Clauset et al. 2004) method utilizes both modularity and hierarchical agglomerative approaches. It is also called the fast greedy one due to a standard greedy way and is significantly quicker than other algorithms. It starts with each node in its community and joins the communities that introduce the most significant modularity increase at each step. The procedure is repeated until no such pair exists.

The Girvan–Newman (GN) (Girvan & Newman 2002) method identifies communities by iteratively removing edges from the original graph. It takes an edge based on its score that, in most cases, the edge with the most significant betweenness centrality value is taken at each step.

Louvain's (LV) (Blondel et al. 2008) method maximizes a modularity score for each community. It is done in two steps: local nodes moving and network aggregation. Each node is transferred to the community that yields the most significant impact on the quality function. Afterward, an aggregated network is created utilizing the partitions computed in the first step. Each community in this partition becomes a node in the aggregate network. The procedure is finished when the quality function (modularity) cannot be further improved.

The Leiden (LN) (Traag et al. 2019) method is an improvement of the Louvain algorithm. It consists of three phases: local moving of nodes, partition refinement, and network aggregation based on the refined partitions. The non-refined division is used to create an initial partition for the aggregate network.

The label propagation (LP) (Cordasco & Gargano 2011) method identifies node groups utilizing only the network structure. It does not need a pre-defined objective function or prior information about the communities. According to the following flow, communities are discovered: Each node gets a unique label—some identifier; then, the simulation is carried out. Each node updates its label to the most popular among the neighbors at each iteration. The procedure stops when each node has the majority label of its neighbors. It is not as deterministic as each time and can provide different results. Multiple simulations should be done, and the most popular division should be used.

Walktrap (WP) (Pons & Latapy 2005) utilizes random walks to detect communities in a network. It is based on the idea that the walks are more likely to stay within the same community because only a few edges lead outside a given community. It conducts short random walks and uses a hierarchical agglomerative approach to merge separate communities bottom-up.

The belief propagation (BF) (Zhang & Moore 2014) community detection method tries to obtain a consensus of many high-modularity partitions. It is achieved by utilizing a scalable message-passing algorithm based on the modularity metrics treated as a Hamiltonian and applying the cavity method.

Infomap (IP) (Rosvall & Bergstrom 2008) is based on information theory. It uses the random walk probability flow on a network as a proxy for information flows in the real system. That information divides the network into modules, compressing the probability flow description.

GA (Pizzuti 2008) is a genetic-based method to find communities in networks. It detects communities by structure, classifying densely connected nodes into a group. It optimizes a productive but straightforward fitness function to identify densely connected groups of nodes with sparse connections between groups.

Gemsec (GM) (Rozemberczki et al. 2019) is based on a graph embedding algorithm that learns a clustering of the nodes simultaneously with computing their embedding. It places nodes in an abstract feature space where the vertex features minimize the negative log-likelihood of preserving sampled vertex neighborhoods. It also incorporates known social network properties through a machine learning regularization.

Kcut (Ruan & Zhang 2007) is a spectral-based algorithm for community detection. It is helpful for undirected and non-overlapping social networks. It provides a unique combination of recursive partitioning and direct k-way methods, guaranteeing the efficiency of a recursive approach while also having the same accuracy as a direct k-way method.

The Markov clustering algorithm (MCL) (Enright 2002) utilizes simulation stochastic-based flow in graphs. It discovers clusters with a mathematical bootstrapping procedure. It computes random walk probabilities through the network and merges them using two transforming operations: expansion and inflation. It is achieved utilizing Markov (stochastic) matrices that contain the mathematical concept of random walks on a graph.

Paris (PS) (Bonald et al. 2018) is a hierarchical graph clustering algorithm inspired by modularity-based clustering techniques. It uses a distance between clusters induced by the probability of sampling node pairs to follow up the agglomerative approach to merge communities. The algorithm's output is a regular dendrogram, which reveals the multi-scale structure of the graph.

Spinglass (SPS) (Reichardt & Bornholdt 2006) relies on an analogy between Potts spin glass’s viral statistical mechanic model and the community structure. The network’s community structure is interpreted as the spin configuration that minimizes the energy of the spin glass, with the spin states being the community indices. It applies the simulated annealing optimization technique to optimize the modularity.

Surprise (SRC) (Traag et al. 2015) method to discover communities is based on a dedicated metric to evaluate them called a surprise. This quality metric assumes that edges between vertices emerge randomly according to a hypergeometric distribution. Partitions get a better score if it is less likely to result from a random realization.

Scalable Community Detection (SCD) (Prat-Pérez et al. 2014) is a disjoint community detection algorithm combining different strategies. It partitions the graph by maximizing the Weighted Community Clustering (WCC). Firstly, clusters are built around highly clustered nodes, and then the initial partition is refined using the approximate WCC.

SBM DL (SBM) (Peixoto 2014) extends the stochastic block model (SBM) approach to finding communities. It utilizes Monte Carlo and the greedy heuristic to infer the stochastic block model. It fits the non-overlapping stochastic block model by description length minimization with an agglomerative heuristic.

## Simulation environment

The presented research has been carried out with RPaSDT (Rumour Propagation and Source Detection Toolkit) (Frąszczak 2022). It is an open-source toolkit available with an MIT license that simulates and analyzes the accuracy of the most common source detection methods. It is based on Python and well-known computing libraries. It provides a highly configurable, easily reusable, and user-friendly GUI-based application to simulate and analyze real scenarios for source detection problems. It allows the preparation of a rumor propagation experiment under any network topology, along with the well-known literature diffusion models, and identifies potential diffusion sources based on the propagation graph. It is worth mentioning that the described toolkit provides a set of additional tools to perform sophisticated network analyses to select different sources and verify how the diffusion under a given topology and origins set could behave. The software can also simulate propagation and source detection for other domains like epidemics or virus detection. This propagation can be manufactured with available models in the toolkit and the source identification process with known methods. It is worth mentioning that a set of auxiliary graph analysis tools includes various available community detection methods. The said properties make it easy to simulate and analyze the wide range of source detection methods utilizing different community detection methods under other conditions.

**Fig. 1:**
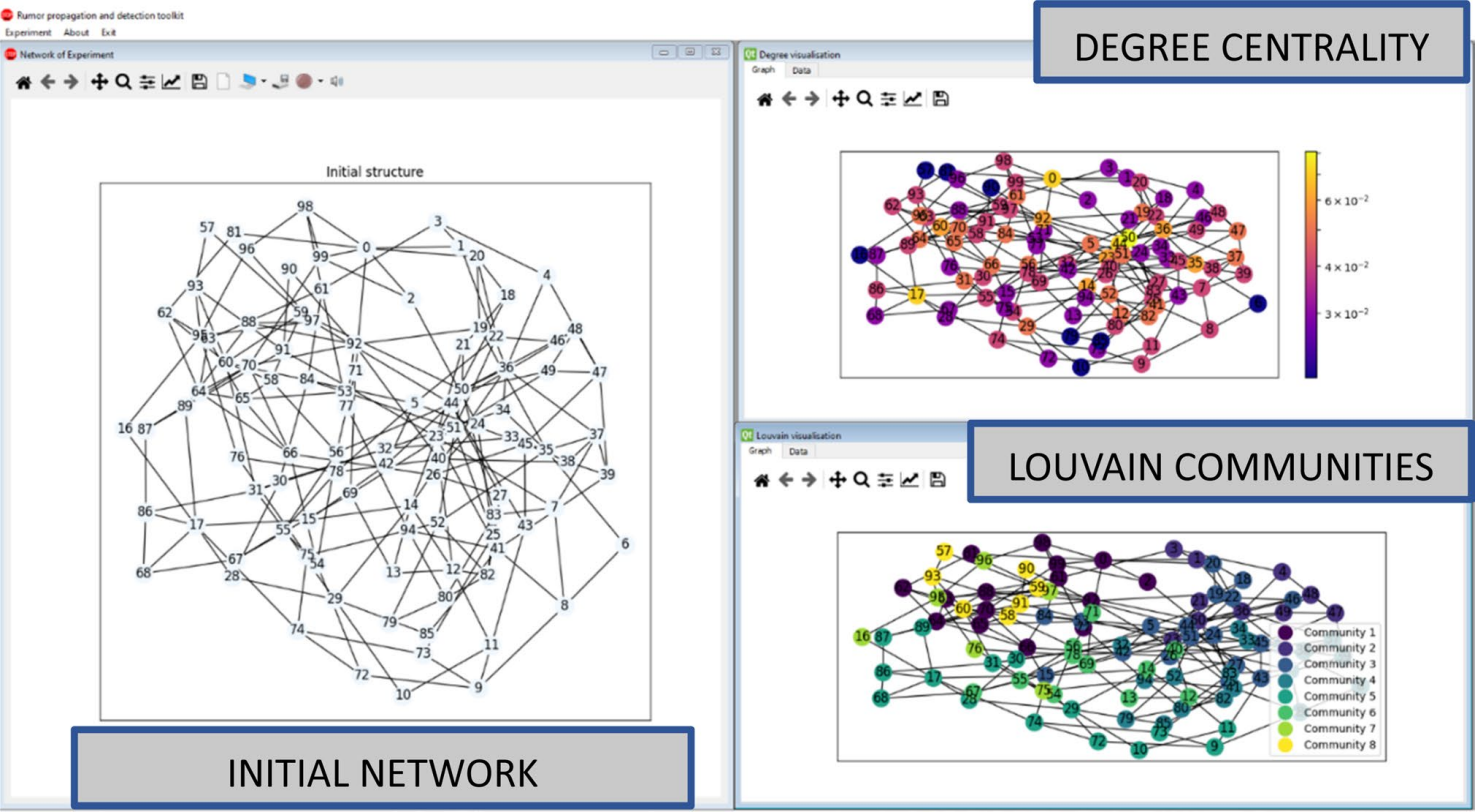
RPaSDT use case—network analysis: initial network structure, degree centrality, and Louvain communities analysis

**Fig. 2:**
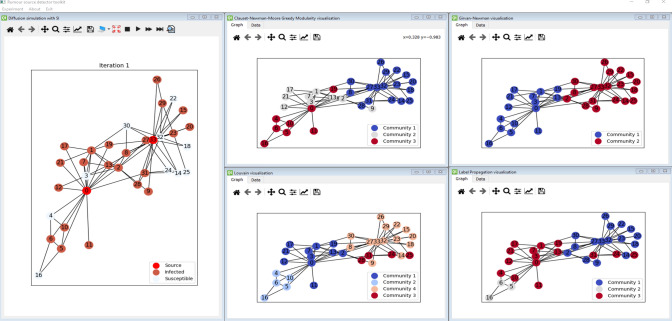
RPaSDT—visualization of different community detection methods for the given infection graph

The simulation environment has been implemented in a window-based approach to visualize and manage different analysis aspects simultaneously. Each window contains a separate model, which does not affect the others. It means that the user can run multiple separate analyses experiments on the initial network structure and compare them, as presented in Fig. 1. All windows can be rearranged any way, making the analysis easier. Each window has a separate toolbar that provides different operations to carry out dedicated functions for the window context. In the presented example the software displays three windows: the initial network structure, degree analysis, and performed community detection with Louvain algorithms. The presented scenario is the basic step in the classical network analysis task. Fig. 2 presents further analysis of the introduced network. On the right side, the result of the four community detection methods is presented, whereas the left one presents the results of the simulation of the rumor propagation initiated by nodes ‘0’ and ‘33.’

## Simulation conditions

Simulation experiments were conducted to assess the accuracy of the well-known community detection methods in the literature that have not been applied yet to find rumor outbreaks in online social networks. This issue is essential due to the possibility of using the divide and conquer rule to divide the task of finding multiple sources in the network. As presented in the previous sections, this area of interest has not been well studied yet. The simulation case study has been carried out with multiple scenarios. The simple one is described in detail, and only simulation results are presented graphically with RPaSDT. The sources are chosen according to their centrality metrics that indicate the most valuable nodes in information propagation (Britt et al. 2021; Das & Kumar Sinha 2018; Frąszczak 2021b). For all cases, the node betweenness centrality metric is used.

**Fig. 3:**
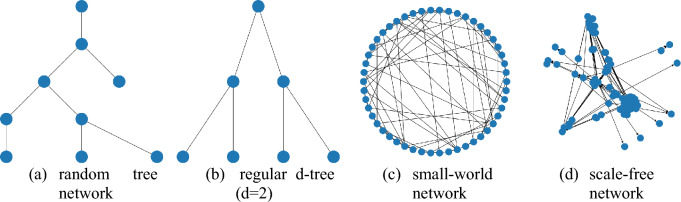
Different network topologies: **a** random tree, **b** regular d-tree, **c** small-world, **d** scale-free network

The dataset used in the analysis includes both synthetical and real-world networks. Synthetical ones have been generated according to some scheme. The most popular social network analyses are small-world (SW), scale-free (SF), including Barabasi–Albert (BA) and Watts–Strogatz (WA), and Erdos–Renyi (ER) (Frąszczak 2021a; Shelke & Attar 2019). The graphical representation of the mentioned networks presents Fig. 3. Moreover, tree-based networks are also used for the rumor source detection problem. For the simulation, SW and BA networks are used, sequentially representing uniform and non-uniform networks, where nodes represent the real individuals, and the edges represent the connection between them in the network. The networks are generated based on the (Ju et al. 2022) used for COVID-19 rumor propagation. WS is generated with *p* = 0.4 and *k* = 10, whereas BA with *k* = 10, *m* = 5.

Real-world datasets have been built upon real social network analysis. A dedicated tool was often prepared to get all the necessary data from the most popular social platforms. Real-world datasets mainly come from Twitter, Facebook, Wiki-vote, Chinese microblogging, Sina Weibo, and Enrol Email for rumor source detection. The datasets described below were used (Ryan & Nesreen 2015; Shu et al. 2019; *Stanford Large Network Dataset Collection*, b.d.). The research was conducted for different types of networks to determine their pros and cons based on network structural properties. The properties of the networks used in the study are presented in Table 3.

**Table 3:** Networks and their analysis used in the study

Network	Nodes	Edges	Density	Assortativity	Avg. clustering coefficient	Degree (min/avg/max)
Karate	34	78	0.1390	− 0.4756	0.5706	1/4/17
Football	115	613	0.0935	0.1624	0.4032	7/10/12
SF-1	500	2475	0.0198	− 0.0966	0.0659	5/9.9/69
SM-1	500	2500	0.0200	− 0.0244	0.1640	5/10.0/16
SF-2	1000	4975	0.0100	− 0.0613	0.0423	5/9.95/126
SM-2	1000	5000	0.0100	− 0.0061	0.1478	5/10.0/16
Facebook	4039	88,234	0.0108	0.0636	0.6055	1/44/1045
Social	12,600	671,000	0.0008	− 0.1219	0.2275	1/10/8700

**Fig. 4:**
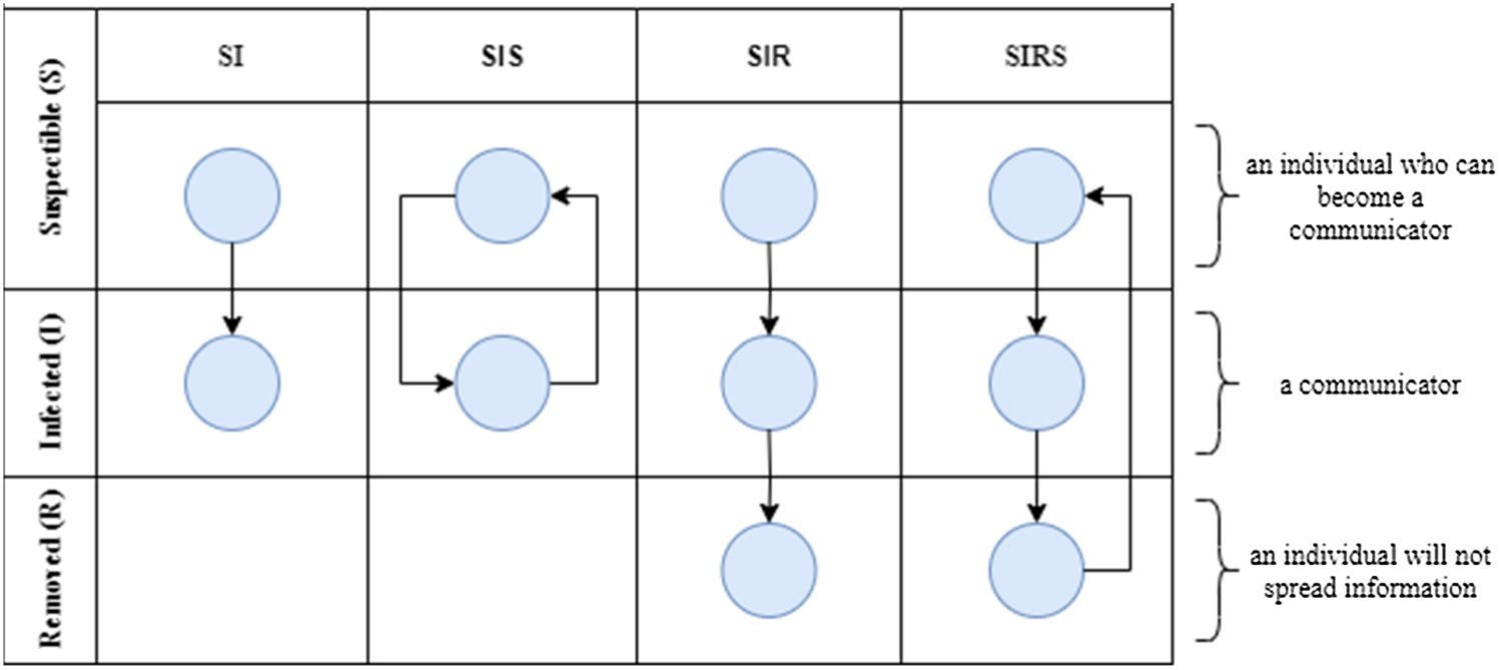
Epidemic models in information spread context

Rumor diffusion in social media platforms can propagate in various ways, but this process’s aim is always the same: to cover as many nodes of the networks as soon as possible. The researchers have developed various models to simulate different behaviors by utilizing the gathered data and analyzing past events. Nowadays, the most famous and willing applied for multiple domains are epidemic models. They have been developed based on an epidemic spread analysis in society. They utilize compartments, mutually exclusive groups based on their disease status. Each individual is located in one compartment at a given time but can move to another depending on the model parameters. There are two main hypotheses around this approach: Each node can be classified into a distinct state (compartment), and each individual has the same opportunity to meet an infected node. It is proven that information spread via online social networks can follow the same rules. The simulation propagation is conducted with the SIR model as it better imitates the rumor diffusion in online social networks. This approach allows nodes to get “healed” and stop the propagation after realizing that passed information is malicious. A summary of the most popular ones is presented in Fig. 4 (Cheng et al. 2013; Kasprzyk et al. 2011; Kasprzyk & Najgebauer 2021; Mei Li et al. 2017).

The SIR model has been used for the simulation with the configuration presented in (Ju et al. 2022). This configuration was used for rumor propagation of COVID-19 fake information. As introduced in that paper, such a model is still eligible to simulate rumor diffusion in social networks. The probability of the node getting “infected” (transition from S to I state) is 0.1, and the likelihood of the node stopping propagation (transition from I to R state) is 0.05 too. The primary purpose of this research is to measure the accuracy of the network partitioning methods to identify rumor outbreaks. The infection graph should be connected, which would be hard to achieve considering only currently infected nodes. The infection graph is computed on nodes either in the infected or recovered state.

## Simulation

Rumor propagation simulation has been conducted with data sources described in Table 3 with the SIR model. The infection graph $G_{I}$ is computed based on both recovered and infected nodes. The simulation is examined for the number of source nodes representing 0.1%, 1%, and 10% of all nodes in the network, respectively. The node betweenness centrality metric indicates the best ones (Şen et al. 2016), and 50 iterations simulate the process. That number can be exceptionally increased to obtain an infected-connected graph. The detection process could be disturbed if some communities were disconnected and initially indicated. The case with 100% network coverage is omitted as it would make it harder to identify real sources in the given network, as the whole network would be analyzed. This approach has some real-case scenario premises, as in general, fake news is not propagated across the entire network, only it’s part. After simulating the expected number of rumor diffusion interactions, community detection, and network partitioning algorithms are applied to the given $G_{I}$. Their accuracy is evaluated, and then utilization of the well-known source detection methods is also verified (Table 4).

**Table 4:** Metrics used for the evaluation of rumor outbreaks detection

Name	Formula	Analysis
Average detection error (ADE)	${\text{ADE}} = \frac{{\sum\nolimits_{N}^{i = 1} {ABS(\left| {\{ {\text{retrieved}}\,{\text{outbreaks}}_{i} \} } \right| - \left| {\{ {\text{true}}\,{\text{outbreaks}}_{i} \} } \right|)} }}{N}$ where $N$- the number of experiments	The ADE is an average ratio of the difference between the number of detected and true rumor outbreaks
Precision	${\text{Precision}} = \frac{{\left| {\{ {\text{retrieved outbreaks}}\} \cap \{ {\text{true outbreaks}}\} } \right|}}{{\left| {\{ {\text{retrieved outbreaks}}\} } \right|}}$	Precision is the ratio of the number of correctly identified outbreaks and overall retrieved outbreaks
Recall	${\text{Recall}} = \frac{{\left| {\{ {\text{retrieved outbreaks}}\} \cap \{ {\text{true outbreaks}}\} } \right|}}{{\left| {\{ t{\text{rue outbreaks}}\} } \right|}}$	Recall is the ratio of the number of correctly identified outbreaks over the real true outbreaks
*F*-measure	$F - {\text{measure }} = \frac{{2 \times {\text{precision}} \times {\text{recall}}}}{{{\text{precision}} + {\text{recall}}}}$	*F*-measure is the ratio of correctly found outbreaks to the sum of all testing outbreaks

**Fig. 5:**
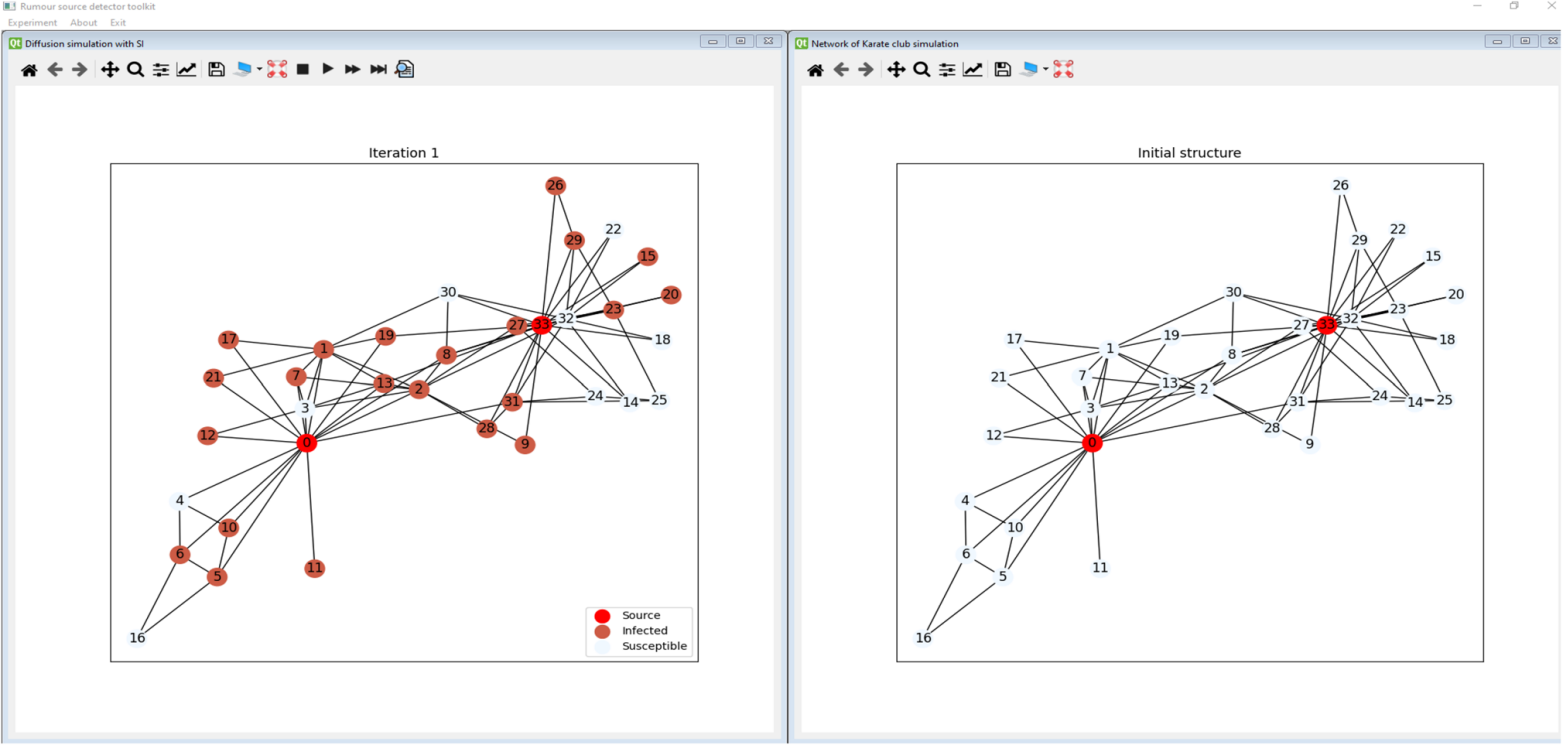
Initial network with selected sources and rumor propagation over it

**Fig. 6:**
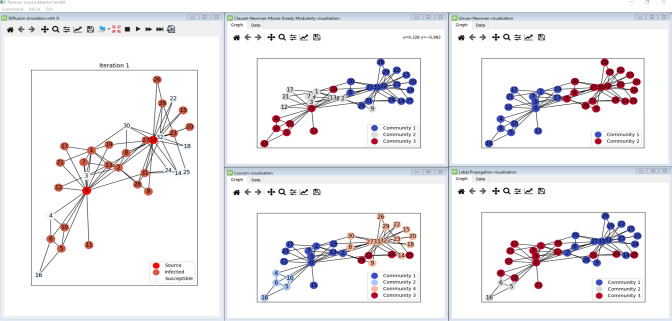
Visualization of the rumor outbreak detection based on infection graph with different methods

In the simplest case, the Karate club network is analyzed and visualized. Only the simulation results are presented for the other scenarios, as for most networks, it is hard to picture them due to their size. It is a social network of a university Karate club. More about that network can be found in Table 3. Firstly, the expected number of sources with the selected method is selected, e.t. nodes 2 and 33. Then the rumor propagation process is simulated by the expected number of iterations with the SIR model. The obtained infection graph $G_{I}$ is then analyzed with rumor outbreak detection methods. The described flow is presented in Figs. 5 and 6. The first one presents the initial structure—the right part and the situation after simulating the propagation from the source nodes, whereas Fig. 6 presents the results of the outbreak detection methods. It can be noticed that the visual analysis for all the methods can be performed simultaneously.

Tables 5, 6, 7, 8 present the results of each algorithm execution under different networks. The summary shows the detected communities, their size, and computational time. The table contains data averaged over ten experiments per case to remove a random bias. The simulation was performed with Intel Core i7-10,700 CPU 2.90 GHz, supported by 64 GB RAM and SSD disk running on the Linux Ubuntu platform. The analysis was performed on the host machine with the runnable package of RPaSDT (Frąszczak 2022). It also provides a Docker-based runtime environment, but the standalone package dedicated to the Linux platform was used to omit any redundant load. Computational time was rounded up to a decimal part of the second. The results for the algorithms, which took over 120 s, are omitted as they would not be a good fit to solve such problems for much bigger networks containing billions of nodes. Moreover, they could be ineffective for processing networks in real time (Table 9).

**Table 5:** Simulation results for Karate and Football real social networks

	Karate									Football								
Method	Sources																	
	0.01%—2, |IG|= 13, Cov = 36%			0.1%—3, |IG|= 17, Cov = 50%			1%—4, |IG|= 23, Cov = 68%			0.01%—2, |IG|= 30, Cov = 26%			0.1%—6, |IG|= 72, Cov = 62%			1%—12, |IG|= 23, Cov = 92%		
	Detected outbreaks/min | avg | max outbreak size/computation time(s)																	
LV	3	4|8|11	0.1	3	4|6|7	0.1	3	2|4|6	0.1	5	3|6|9	0.1	9	5|8|11	0.1	9	7|12|22	0.1
BF	8	1|3|8	8.6	6	1|3|4	8.3	5	1|2|5	8.2	2	12|15|18	7.8	4	13|18|27	2	6	8|18|24	3
LN	3	4|8|11	0.1	3	4|6|7	0.1	3	2|4|6	0.1	5	3|6|9	0.1	9	5|8|11	0.1	10	7|10|14	0.1
LP	3	2|8|18	0.1	2	2|8|15	0.1	1	12|12|12	0.1	6	3|5|9	0.1	10	3|7|20	0.1	10	4|10|22	0.1
CNM	3	4|8|11	0.1	3	4|6|7	0.1	3	2|4|6	0.1	5	3|6|9	0.1	7	6|10|17	0.1	5	18|21|25	0.1
GN	2	10|12|13	0.1	2	8|8|9	0.1	3	2|4|6	0.1	6	3|5|8	0.1	5	7|14|26	0.1	7	10|15|30	0.1
GA	3	5|12|16	3.6	5	2|3|6	3	2	10|11|12	2.8	7	3|4|7	4.1	7	6|10|19	7.3	13	2|8|19	10.2
IP	3	4|8|11	0.1	2	7|8|10	0.1	1	12|12|12	0.1	7	2|4|7	0.1	8	5|9|12	0.1	9	8|12|15	0.1
Kcut	3	1|8|21	0.1	4	1|4|14	0.2	2	1|6|11	0.1	7	1|4|24	0.2	5	1|14|68	0.9	8	1|13|98	1.3
MCL	2	9|12|14	0.1	2	7|8|10	0.1	1	12|12|12	0.1	7	2|4|7	0.1	12	1|6|11	0.1	12	5|9|13	0.1
PS	2	8|12|15	0.1	2	6|8|11	0.1	4	2|3|4	0.1	6	3|5|7	0.1	6	6|12|17	0.1	2	51|52|54	0.1
SPS	4	3|6|8	0.1	3	4|6|7	0.1	4	2|3|4	0.1	6	3|5|7	0.1	12	1|6|11	0.2	11	6|10|14	0.2
SRC	8	1|3|6	0.1	5	1|3|6	0.1	6	1|2|3	0.1	7	2|4|7	0.1	12	2|6|10	0.1	12	5|9|13	0.1
WP	3	5|8|13	0.1	2	7|8|10	0.1	3	1|4|6	0.1	6	3|5|8	0.1	10	4|7|11	0.1	10	7|10|14	0.1
SCD	2	7|12|16	0.8	2	7|8|10	0.4	3	2|4|5	0.2	2	13|15|17	0.7	2	29|36|43	3.9	2	44|52|61	22.4
SBM	1	23|23|23	0.1	1	17|17|17	0.1	1	12|12|12	0.1	2	12|15|18	0.1	8	6|9|12	0.1	10	7|10|14	0.1

**Table 6:** Simulation results for Social and Facebook real social networks

	Social									Facebook								
Method	Sources																	
	0.01%—13, |IG|= 6387, Cov = 50%			0.1%—127, |IG|= 6715, Cov = 54%			1%—1260, |IG|= 7094, Cov = 56%			0.01%—5, |IG|= 105, Cov = 88%			0.1%—41, |IG|= 3817, Cov = 94%			1%—404, |IG|= 3831, Cov = 95%		
	Detected outbreaks/min | avg | max outbreak size/computation time(s)																	
LV	16	3|420|1620	1.8	17	2|417|1817	1.2	28	3|228|1594	1.1	16	17|223|527	1.3	16	19|239|502	2	16	19|239|509	1.3
BF	N\A	N\A	N\A	N\A	N\A	N\A	N\A	N\A	N\A	N\A	N\A	N\A	N\A	N\A	N\A	N\A	N\A	N\A
LN	18	3|373|1451	0.2	19	3|373|2360	0.1	30	3|213|1575	0.2	15	18|238|526	0.2	16	19|239|519	0.2	16	19|239|509	0.2
LP	32	2|210|6598	0.3	45	2|158|6885	0.3	81	2|79|6059	0.3	28	2|127|995	0.3	34	2|112|999	0.2	33	2|116|998	0.2
CNM	44	2|153|2991	82.7	36	2|197|2137	75.4	72	2|89|1876	69.2	11	6|324|961	15.3	14	6|273|1004	19.4	13	7|295|946	15.6
GN	6	34|1119|3579	0.6	7	4|1013|3796	0.7	4	475|1597|2297	0.7	16	9|223|569	1	18	1|212|569	1.2	18	1|213|572	1.2
GA	N\A	N\A	N\A	N\A	N\A	N\A	N\A	N\A	N\A	N\A	N\A	N\A	N\A	N\A	N\A	N\A	N\A	N\A
IP	218	2|31|1701	0.3	249	2|28|2166	0.3	273	2|23|2880	0.4	6	11|594|1274	0.4	7	35|545|1268	0.4	6	190|638|1308	0.4
Kcut	N\A	N\A	N\A	N\A	N\A	N\A	N\A	N\A	N\A	N\A	N\A	N\A	N\A	N\A	N\A	N\A	N\A	N\A
MCL	437	1|15|3735	2.5	455	1|16|3813	2.6	577	1|11|2334	2.2	9	12|396|991	1.7	10	29|382|990	1.7	10	40|383|996	1.7
PS	2	2313|3358|4402	4.4	2	53|3547|7041	5.2	3	51|2129|4090	2.3	5	225|713|1275	1.3	7	33|545|1313	1.3	7	48|547|1339	1.4
SPS	19	2|353|1566	112.1	N\A	N\A	N\A	22	3|290|1709	112.4	N\A	N\A	N\A	N\A	N\A	N\A	N\A	N\A	N\A
SRC	3449	1|2|796	0.2	3634	1|2|806	0.2	2558	1|2|736	0.2	281	1|13|298	0.2	313	1|12|298	0.2	322	1|12|300	0.2
WP	2349	1|3|1528	3.6	235	1|30|2888	3.9	1981	1|3|1867	3.2	51	2|70|464	1.7	61	2|63|465	1.8	64	2|60|457	1.8
SCD	N\A	N\A	N\A	N\A	N\A	N\A	N\A	N\A	N\A	N\A	N\A	N\A	N\A	N\A	N\A	N\A	N\A	N\A
SBM	42	1|160|2671	7.5	35	3|203|2892	7.2	40	3|160|2187	7.3	59	1|60|175	4.2	66	1|58|178	5.5	64	1|60|175	4.6

**Table 7:** Simulation results for Small-World synthetical social networks

	SM-1									SM-2								
Method	Sources																	
	0.01%—2, |IG|= 105, Cov = 11%			0.1%—5, |IG|= 3817, Cov = 48%			1%—50, |IG|= 3831, Cov = 89%			0.01%—2, |IG|= 112, Cov = 12%			0.1%—10, |IG|= 415, Cov = 42%			1%—100, |IG|= 896, Cov = 90%		
	Detected outbreaks/min | avg | max outbreak size/computation time(s)																	
LV	6	5|9|14	0.1	15	7|16|29	0.1	13	21|34|53	0.1	12	6|9|18	0.1	17	9|24|47	0.1	20	26|45|72	0.1
BF	2	26|28|29	8.8	6	34|41|48	7.8	6	63|74|103	33.8	3	32|37|48	10.6	7	49|59|82	19.8	N\A	N\A	N\A
LN	6	5|9|14	0.1	15	7|16|22	0.1	13	16|34|53	0.1	10	8|11|19	0.1	17	14|24|44	0.1	22	24|41|85	0.1
LP	8	2|7|18	0.1	22	2|11|62	0.1	2	16|222|428	0.1	26	2|4|21	0.1	46	2|9|38	0.1	36	4|25|355	0.1
CNM	7	5|8|14	0.1	11	7|22|38	0.1	9	9|49|84	0.3	11	7|10|19	0.1	14	11|30|64	0.1	9	17|100|202	1.1
GN	9	1|6|14	0.1	16	1|15|29	0.1	9	23|49|85	0.1	15	1|7|17	0.1	16	1|26|76	0.1	14	26|64|172	0.1
GA	12	2|5|13	10.3	32	2|8|30	63.3	N\A	N\A	N\A	26	2|4|11	32.3	N\A	N\A	N\A	N\A	N\A	N\A
IP	8	3|7|12	0.1	23	3|11|19	0.1	28	7|16|29	0.1	3	17|37|51	0.1	40	3|10|16	0.1	57	4|16|27	0.1
Kcut	8	1|7|48	0.3	8	1|30|237	3.3	8	1|56|437	10	6	1|19|107	1	7	1|59|409	8.2	8	1|112|889	35
MCL	14	1|4|12	0.1	74	1|3|10	0.1	179	1|2|14	0.1	35	1|3|8	0.1	128	1|3|12	0.1	371	1|2|12	0.3
PS	2	26|28|29	0.1	21	5|12|20	0.1	13	14|34|58	0.1	6	8|19|31	0.1	6	24|69|126	0.1	18	20|50|94	0.1
SPS	9	2|6|12	0.2	16	7|15|23	1.7	18	12|25|38	1.6	12	4|9|17	0.6	22	7|19|35	3.2	24	13|37|60	5.8
SRC	16	1|3|9	0.1	36	2|7|14	0.1	44	3|10|17	0.1	26	1|4|9	0.1	61	1|7|15	0.1	79	3|11|18	0.1
WP	7	2|8|17	0.1	19	3|13|26	0.1	16	15|28|86	0.1	23	2|5|13	0.1	29	2|14|75	0.1	34	10|26|188	0.1
SCD	2	27|28|28	2.8	N\A	N\A	N\A	N\A	N\A	N\A	2	22|56|90	31.1	N\A	N\A	N\A	N\A	N\A	N\A
SBM	2	14|28|41	0.1	4	25|61|147	0.2	15	16|30|52	0.6	3	8|37|93	0.1	11	23|38|136	0.4	30	20|30|45	2

**Table 8:** Simulation results for Scale-Free synthetical social networks

	SF-1									SF-2								
Method	Sources																	
	0.01%—2, |IG|= 336, Cov = 67%			0.1%—5, |IG|= 408, Cov = 82%			1%—50, |IG|= 427, Cov = 86%			0.01%—2, |IG|= 789, Cov = 79%			0.1%—10, |IG|= 825, Cov = 83%			1%—100, |IG|= 864, Cov = 86%		
	Detected outbreaks/min | avg | max outbreak size/computation time(s)																	
LV	11	12|31|50	0.1	10	8|41|63	0.1	10	12|43|83	0.1	12	19|66|128	0.2	12	25|69|131	0.2	10	41|86|168	0.2
BF	8	20|42|65	57.7	8	22|51|85	78.4	8	34|53|67	84.7	N\A	N\A	N\A	N\A	N\A	N\A	N\A	N\A	N\A
LN	10	20|34|47	0.1	8	34|51|75	0.1	11	27|39|60	0.1	11	32|72|108	0.1	10	17|82|129	0.1	12	41|72|117	0.1
LP	1	336|336|336	0.1	1	408|408|408	0.1	1	427|427|427	0.1	1	789|789|789	0.1	1	825|825|825	0.1	1	864|864|864	0.1
CNM	9	10|37|77	0.2	8	10|51|86	0.3	9	6|47|89	0.3	11	4|72|149	1	10	14|82|170	1.1	10	18|86|166	1.1
GN	11	1|31|73	0.1	15	1|27|54	0.1	13	1|33|63	0.1	12	1|66|265	0.1	18	1|46|218	0.1	19	1|45|108	0.1
GA	35	2|10|68	104.2	N\A	N\A	N\A	N\A	N\A	N\A	N\A	N\A	N\A	N\A	N\A	N\A	N\A	N\A	N\A
IP	1	336|336|336	0.1	1	408|408|408	0.1	1	427|427|427	0.1	1	789|789|789	0.1	1	825|825|825	0.1	1	864|864|864	0.1
Kcut	3	1|112|334	6.1	7	1|58|402	8.5	6	1|71|422	9.3	2	1|394|788	27.8	3	1|275|823	30.6	2	1|432|863	33.2
MCL	117	1|3|42	0.1	175	1|2|44	0.1	184	1|2|34	0.1	349	1|2|57	0.2	376	1|2|49	0.3	416	1|2|55	0.3
PS	8	20|42|65	0.1	16	13|26|35	0.1	23	8|19|29	0.1	17	28|46|72	0.1	8	64|103|160	0.1	33	13|26|42	0.1
SPS	9	30|37|46	3.6	9	16|45|65	4	9	21|47|65	4.4	9	59|88|104	10.6	9	21|92|112	10.6	11	54|79|92	11.8
SRC	69	1|5|15	0.1	80	2|5|12	0.1	86	2|5|11	0.1	126	3|6|19	0.1	139	3|6|16	0.1	140	3|6|15	0.1
WP	20	2|17|105	0.1	30	2|14|99	0.1	38	2|11|89	0.1	66	1|12|188	0.1	49	2|17|205	0.1	68	2|13|235	0.1
SCD	N\A	N\A	N\A	N\A	N\A	N\A	N\A	N\A	N\A	N\A	N\A	N\A	N\A	N\A	N\A	N\A	N\A	N\A
SBM	1	336|336|336	0.2	1	408|408|408	0.3	1	427|427|427	0.4	1	789|789|789	1.6	1	825|825|825	1.8	1	864|864|864	2

**Table 9:** Outbreak detection methods summary ranking

Method	Ex. 1	Ex. 2	Ex. 3	Ex. 4	Ex. 5	Ex. 7	Ex. 8	Ex. 9	Ex. 10	Rank all	Rank SD
LN	1	1	2	5	1	2	2	6	1	21	11
GN	1	7	1	7	1	1	1	3	2	24	7
LV	1	5	2	8	1	4	3	7	4	35	18
CNM	1	1	2	8	6	3	4	8	3	36	18
LP	1	1	2	10	6	6	6	2	6	40	20
IP	1	1	7	4	6	5	8	5	5	42	23
SRC	1	10	8	1	1	8	5	1	8	43	22
WP	1	8	6	3	1	7	7	4	7	44	25

The evaluation of the accuracy of the well-known network partitioning and community methods in identifying rumor outbreaks has been performed with metrics specific to community partitioning introduced in Table 1. They were extended with classification-based methods presented in Table 4 to provide a better evaluation for rumor outbreak identification. For the assessment, the information about propagation and source infection was used to define ground-truth communities used by metrics.

Based on obtained coverage results, it can be observed that analyzed networks can be divided into two groups based on their topology. Scale-Free (Facebook, Social, SF-1, SF-2) for which even a few sources (but very important) can reach a great network coverage. Adding new rumor sources for such networks does not improve the diffusion process. Small-world (Karate, Football, SM-1, SM-2) like adding more rumor sources makes a difference in the context of better network coverage in a shorter time.

**Fig. 7:**
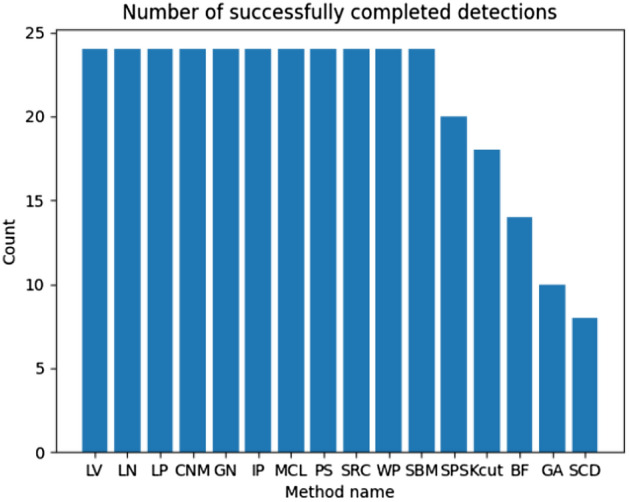
Ex. 1 Completed outbreaks (communities) detection experiments per outbreak detection method (more is better)

Unfortunately, not all examined methods could detect outbreaks in the maximum expected time (the ‘N\A’ value provided in the table), so they were removed from further analysis. The visual representation of the completed detections is presented in Fig. 7. Including them during the research could lead to incorrect conclusions. Moreover, they are not eligible for big networks. Methods removed from the further analysis are SPS, Kcut, BF, GA, and SPL.

**Fig. 8:**
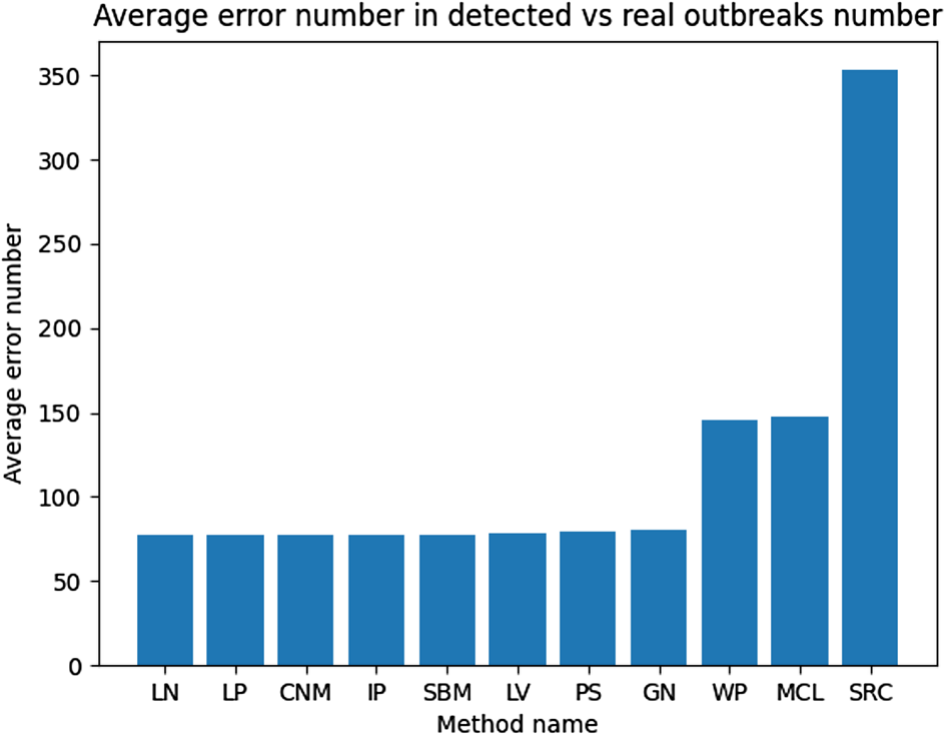
Ex. 2 Average detection error per method (less is better)

**Fig. 9:**
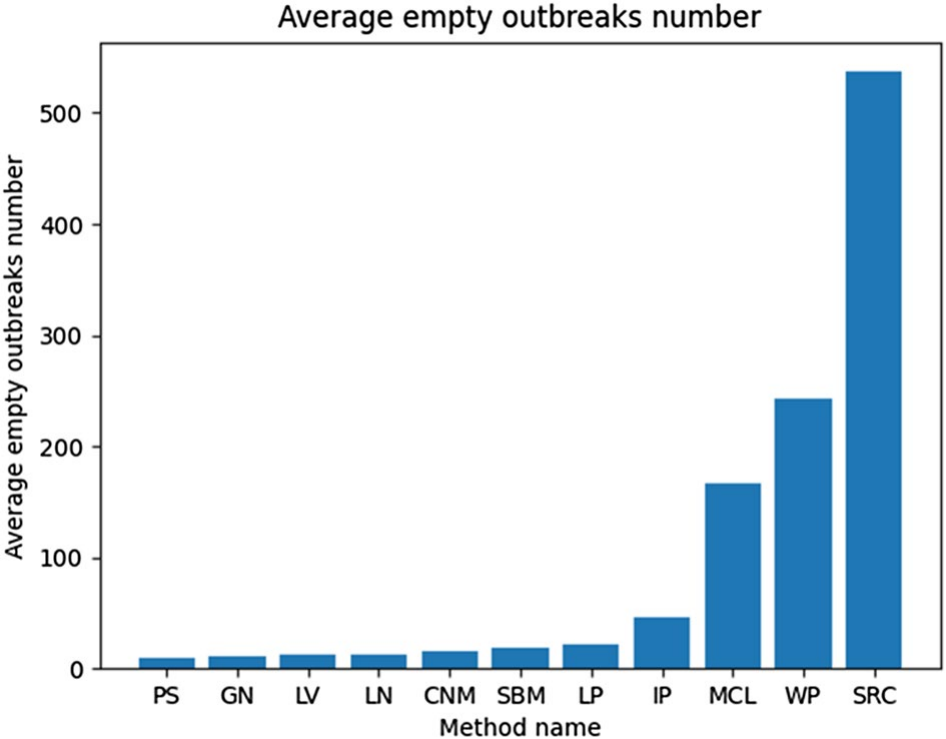
Ex. 3 Average number of identified empty outbreaks per method (less is better)

**Fig. 10:**
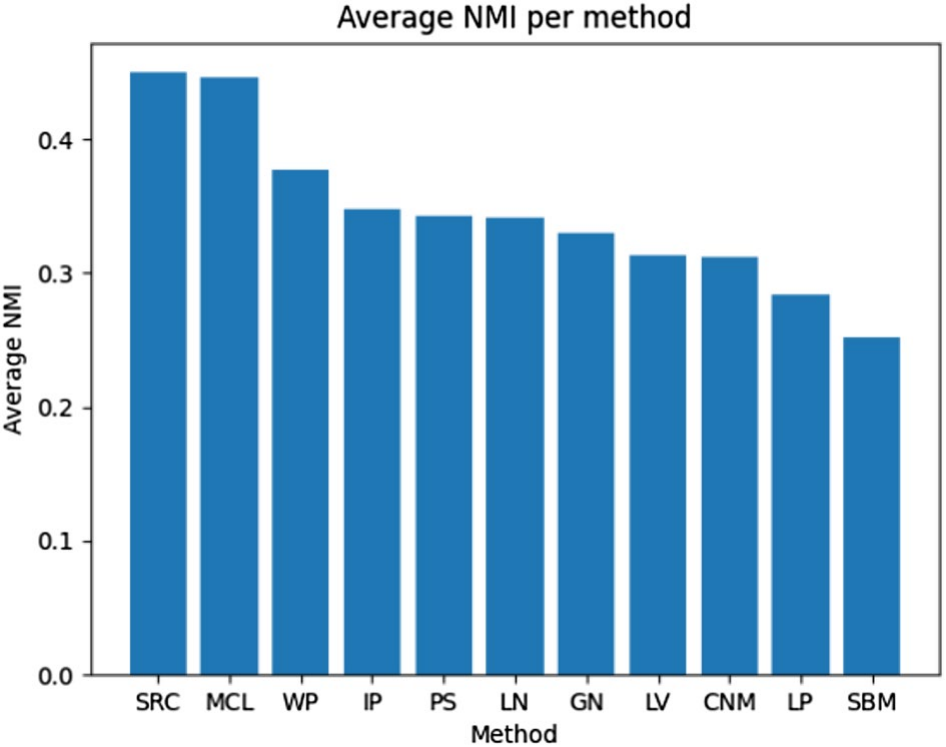
Ex. 4 Average NMI per method (more is better)

In the case of the average detection error presented in Fig. 8, three methods delivered worse results than others, and in the case of the SRC, this difference was significant. The NMI metric for evaluated methods is similar and close to 0.3, but SRC and MCL have more than 0.4 surpassing other approaches. Based on that it looks like only about 30% of all nodes are correctly assigned to a correct outbreak for most of the tested methods. In the context of the rumor source identification, this value is too low as it can lead to inappropriate results or make it harder for such analysis because nodes are assigned to different outbreaks than they belong to. It is also worth mentioning that SRC, MCL, and WP, which are the best in the NMI benchmark, are the worst in ADE—Fig. 8. It means they were better at assigning nodes to correct outbreaks but not so precise in estimating the correct number of them. It can be observed in the above tables that they provided much more groups that are less numerous than other methods causing their NMI to be higher but increasing the error. That thesis is confirmed in Fig. 9 where the SRC method found the biggest number of empty (without the real source) outbreaks which is not good. The best in that metric is PS, although also GN, LV, LN, CNM, SBM, and LP received good results. The well-performed outbreak detection method should have that value as low as possible allowing a reduction in the number of false positives (Fig. 10).

In most cases, the tested techniques overestimate the total number. This trend can be better noticed by removing the biggest outbreaks number from the evaluated dataset. This assumption is confirmed in real-life cases as fake news sources work in small and separate groups (Jin & Wu 2021; Li et al. 2019; Shelke & Attar 2019). What is also worthy of mentioning is that the outcome of correctly identifying the rumor outbreak number is rare and happens mostly for small networks.

For all algorithms, the size of the identified outbreaks varies greatly, often leaving several-node groups with multi-node. Moreover, the difference between the average outbreak size and its outliers is significant.

**Fig. 11:**
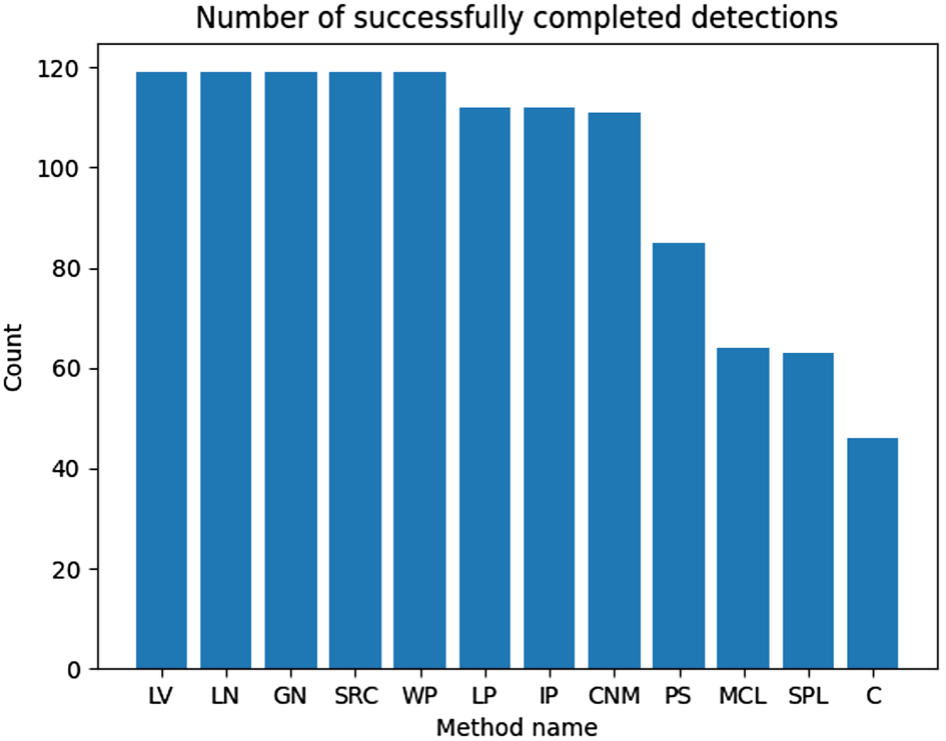
Ex. 5 Completed source detection experiments in identified outbreaks per outbreak detection method (more is better)

**Fig. 12:**
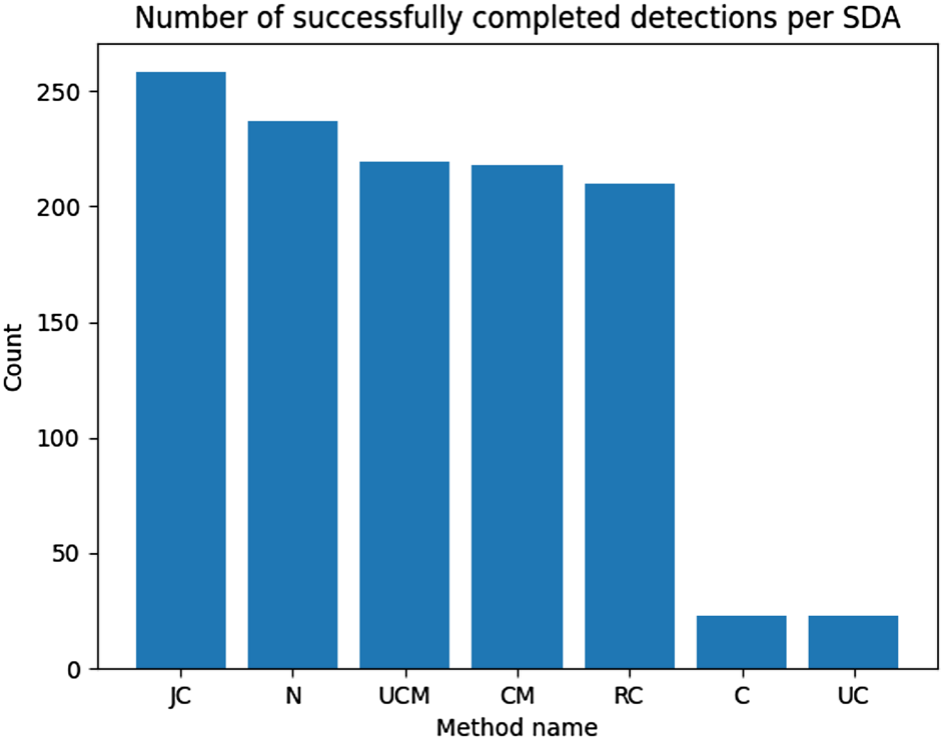
Ex. 6 Completed source detection experiments in outbreaks per source detection method (more is better)

This feature is not expected as it automatically can lead to incorrect results in the context of the rumor source identification. It is unlikely that fake news sources in real scenario started propagating malicious content from an isolated group of users. They aim to reach as many users as possible in the shortest time. In the rumor source identification process, merging such groups with the biggest ones is recommended as it can provide better information context for the detection method. Unfortunately, none of the examined methods has provided results containing outbreaks of similar size. The confirmation of the above thesis will be presented with the effects on the effectiveness of identifying sources further in this paper. Figure 11 illustrates the number of completed source detection tasks per outbreak detection method. It can be observed that only some of them were able to process detection in the expected execution time, which again was set to 120 s. Only some combination of the presented methods can be used to resolve real scenarios with bigger networks. Methods like LP, IP, or CNM could almost perform the expected number of detection besides the ones for the biggest ones. Methods considered for further analysis are SRC, WP, LV, LN, GN, LP, IP, and CNM. The method “C” marked on the figures refers to standard betweenness centrality, “UC” unbiased version, “CM” traditional betweenness centrality based on outbreaks, and “UCM” unbiased version based on outbreaks. Based on the results presented in Fig. 12, it can be noticed that each source detection algorithm has been evaluated with a different number of successes, so further evaluation is conducted per source detection method. Moreover, it can be observed that providing the rumor outbreaks detection part to source detection based on the centralities makes such methods faster as they were able to perform more experiments.

**Fig. 13:**
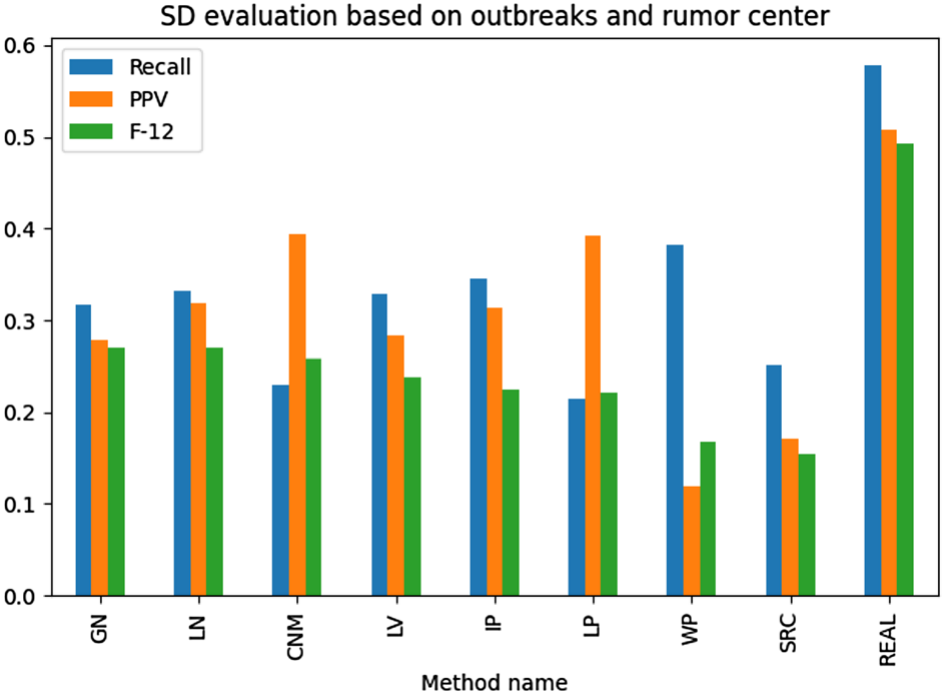
Ex. 7 Source detection evaluation based on Rumor Center with outbreak detection methods

**Fig. 14:**
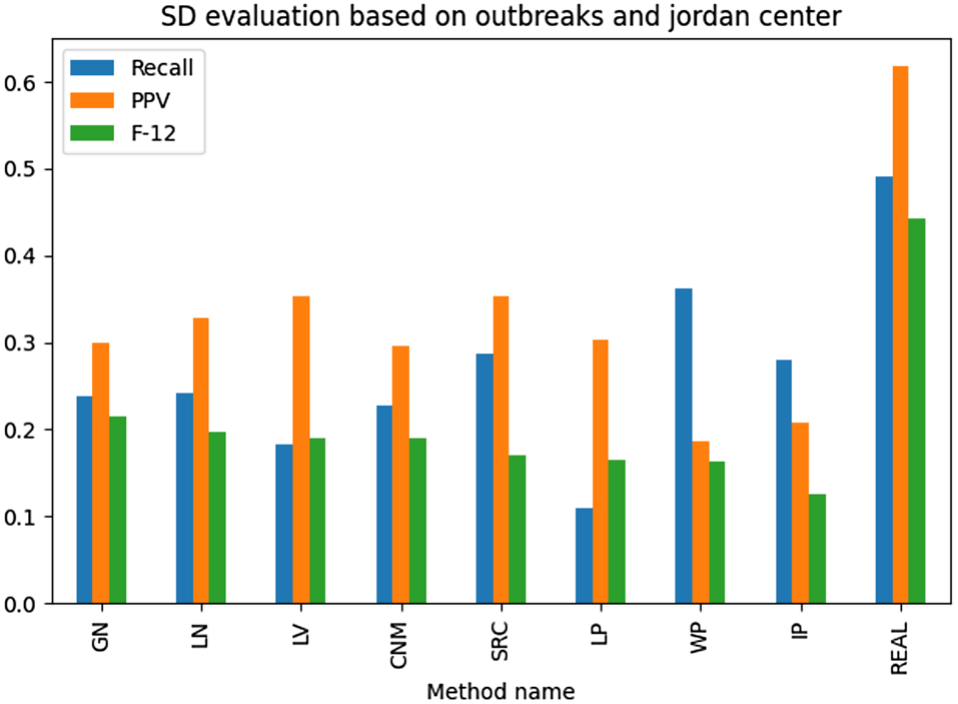
Ex. 8 Source detection evaluation based on Jordan Center with outbreak detection methods

**Fig. 15:**
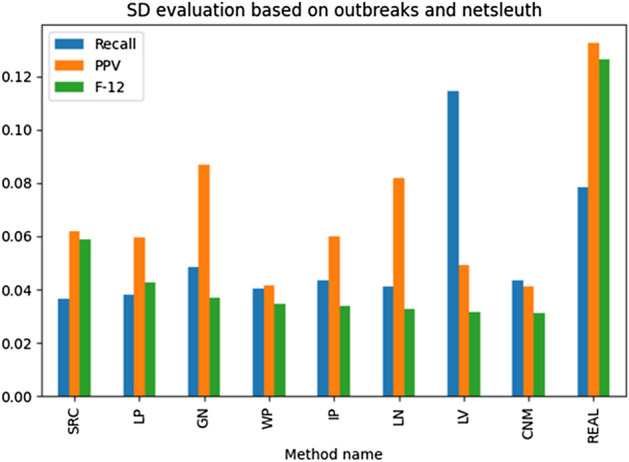
Ex. 9 Source detection evaluation based on NetSleuth with outbreak detection methods

**Fig. 16:**
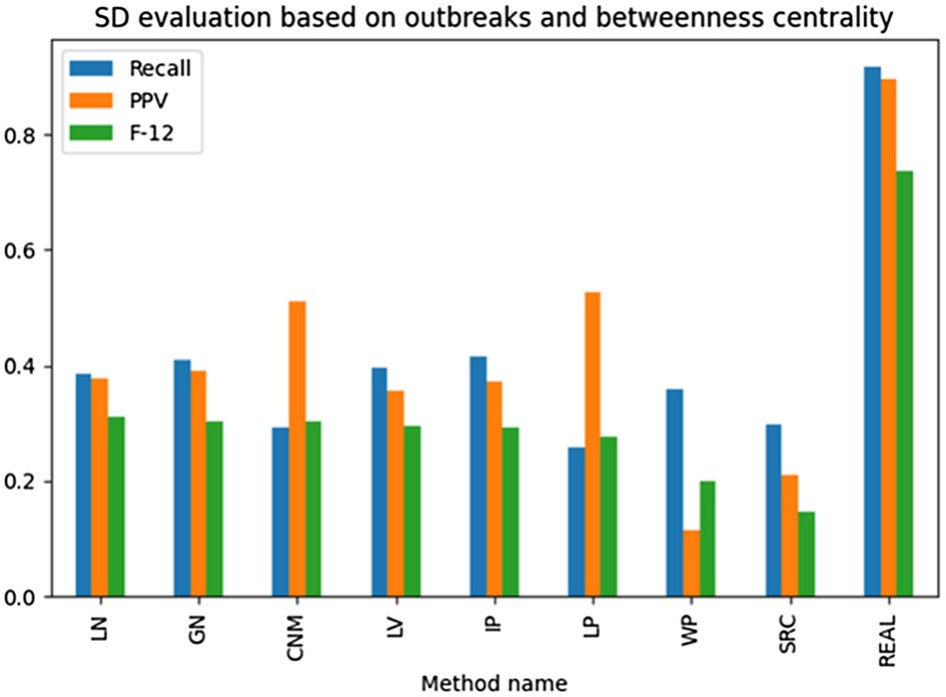
Ex. 10 Source detection evaluation based on betweenness centrality with outbreak detection methods

The results illustrated in Figs. 13, 14, 15, 16 present the examined source detection methods working on outbreaks identified by different techniques. The results are sorted decreasingly based on the *F*-score being a harmonic mean of the precision and recall. Each graph's last column—“REAL”—contains the source detection results found on the real outbreak. The best results are obtained by source detection based on betweenness centrality, as this metric was used to select propagation origins. A simple ranking has been used to find the best method for detecting correct outbreaks. The rank is the position sum of the technique in the analyzed metrics, and the method with the lowest ranking value is considered the best one. In other words, this measure shows how many given algorithm has been the best among the others. Based on the introduced metric, the Leiden algorithm was the best based on all analyses, but GN was the best for the only source detection experiments. Summarizing both LN and GN methods overperformed the other methods and should be taken into further analysis and used as the reference for the new methods.

## Conclusions

Social media platforms are broadly used to exchange information by milliards of people worldwide. Unfortunately, they are increasingly used with malicious intent. Finding a rumor source is a crucial attempt at controlling, preventing, and learning about the propagation of falsified information in networks. Unfortunately, the networks' size and complex structure make the problem of correctly identifying real sources harder. The current research in rumor source detection methods is mostly oriented toward single-source issues, which is inappropriate for real-life scenarios. Most available multi-source detection approaches require the exact number, whereas such information is not generally known a priori. To alleviate the mentioned problem, the presented paper has been introduced. To the best of our knowledge, this is the first comprehensive survey and analysis that focuses on the techniques of seeking propagation outbreaks in various networks without the exact number of them. It presents a variety of well-known network partitioning and community detection methods applied in a simulation case study to identify the best ones and provide their drawbacks and guidelines for new ways straightly oriented toward rumor multi-source identification problem.

The presented results highlight the issue of the lack of methods that can estimate the real number of sources correctly. All of the techniques were designed to use in another context than rumor source detection, with specific conditions. Moreover, the NMI metrics results indicate that only a small part of nodes are correctly assigned to a correct outbreak. Most of the presented methods share the same trend—they overestimate the total number of potential sources, leaving small groups of nodes with huge ones that strongly impact the accuracy of the source detection methods. Another drawback of them is the fact of detecting “empty” outbreaks that do not contain the real source. Moreover, evaluating the source detection methods applied to over-identified outbreaks confirmed the assumption that correct outbreak detection is crucial in finding many sources. The accuracy of all examined source detection methods increased significantly after applying them to the actual propagation outbreaks. However, the various network partitioning approaches gave a great overview, indicating the best ones that should be used in future research. Detecting fake news outbreaks has different properties than network partitioning and requires reliable methods to improve the accuracy of the current procedures. It should be able to recognize the most significant areas of propagation that should be consumed by detection methods to improve their accuracy. Moreover, it should be characterized by short computation time and be eligible for use in real-life scenarios for networks with huge nodes.

## References

[CR1] Ali SS, Anwar T, Rizvi SAM (2020). A revisit to the infection source identification problem under classical graph centrality measures. Online Social Networks and Media.

[CR2] Blondel VD, Guillaume J-L, Lambiotte R, Lefebvre E (2008). Fast unfolding of communities in large networks. J Stat Mech: Theory Exp.

[CR3] Bonald T, Charpentier B, Galland A, Hollocou A (2018) Hierarchical graph clustering using node pair sampling. http://arxiv.org/abs/1806.01664

[CR4] Britt BC, Hayes JL, Musaev A, Sheinidashtegol P, Parrott S, Albright DL (2021). Using targeted betweenness centrality to identify bridges to neglected users in the Twitter conversation on veteran suicide. Soc Netw Anal Min.

[CR5] Brockmann D, Helbing D (2013). The hidden geometry of complex, network-driven contagion phenomena. Science.

[CR6] Chebotarev P, Gubanov D (2020). How to choose the most appropriate centrality measure? arXiv:2003.01052 * [Physics]*

[CR7] Cheng J-J, Liu Y, Shen B, Yuan W-G (2013). An epidemic model of rumor diffusion in online social networks. The European Physical Journal B.

[CR8] Choi J, Moon S, Woo J, Son K, Shin J, Yi Y (2020a) Information source finding in networks: querying with budgets. ArXiv:2009.00795 * [Cs]*. http://arxiv.org/abs/2009.00795

[CR9] Choi J, Moon S, Woo J, Son K, Shin J, Yi Y (2020b) Rumor source detection under querying with untruthful answers. ArXiv:1711.05496 * [Cs]*. http://arxiv.org/abs/1711.05496

[CR10] Clauset A, Newman MEJ, Moore C (2004). Finding community structure in very large networks. Phys Rev E.

[CR11] Cordasco G, Gargano L (2011) Community detection via semi-synchronous label propagation algorithms. http://arxiv.org/abs/1103.4550

[CR12] Danon L, Díaz-Guilera A, Duch J, Arenas A (2005). Comparing community structure identification. J Stat Mech: Theory Exp.

[CR13] Das K, Kumar Sinha S (2018). Centrality measure based approach for detection of malicious nodes in twitter social network. International Journal of Engineering & Technology.

[CR14] Das K, Samanta S, Pal M (2018). Study on centrality measures in social networks: A survey. Soc Netw Anal Min.

[CR15] *Digital News Report 2016*. (b.d.). Reuters. https://reutersinstitute.politics.ox.ac.uk/sites/default/files/research/files/Digital%2520News%2520Report%25202016.pdf.

[CR16] Dong W, Zhang W, Tan CW (2013). Rooting out the rumor culprit from suspects. IEEE International Symposium on Information Theory.

[CR17] Enright AJ (2002). An efficient algorithm for large-scale detection of protein families. Nucleic Acids Res.

[CR18] Fortunato S (2010). Community detection in graphs. Phys Rep.

[CR19] Frąszczak D (2021a) Fake news source detection—the state of the art survey for current problems and research. In: Proceedings of the 37th international business information management association (IBIMA), pp 11381–11389. 10.6084/m9.figshare.16545675

[CR20] Frąszczak D (2021b). Information propagation in social networks—a simulation case study. In: Proceedings of the 38th international business information management association (IBIMA). Innovation management and information technology impact on global economy in the era of pandemic. Cordoba, Spain

[CR21] Frąszczak D (2022). RPaSDT—rumor propagation and source detection Toolkit. SoftwareX.

[CR22] Girvan M, Newman MEJ (2002). Community structure in social and biological networks. Proc Natl Acad Sci.

[CR23] Guille A, Hacid H, Favre C, Zighed DA (2013). Information diffusion in online social networks: A survey. ACM SIGMOD Rec.

[CR24] Higdon N (2020). The anatomy of fake news: A critical news literacy education.

[CR25] Jiang J, Wen S, Yu S, Xiang Y, Zhou W (2015). K-center: An approach on the multi-source identification of information diffusion. IEEE Trans Inf Forensics Secur.

[CR26] Jiang J, Wen S, Yu S, Xiang Y, Zhou W (2017). Identifying propagation sources in networks: State-of-the-art and comparative studies. IEEE Communications Surveys & Tutorials.

[CR27] Jin R, Wu W (2021) Schemes of propagation models and source estimators for rumor source detection in online social networks: a short survey of a decade of research. ArXiv:2101.00753* [Cs]*. http://arxiv.org/abs/2101.00753

[CR28] Ju C, Jiang Y, Bao F, Zou B, Xu C (2022). Online rumor diffusion model based on variation and silence phenomenon in the context of COVID-19. Front Public Health.

[CR29] Kasprzyk R, Najgebauer A (2021) Experimental environment to model, simulate and analyze contagious diseases as a diffusion process in social networks [Preprint]. In: Review. 10.21203/rs.3.rs-923987/v1

[CR30] Kasprzyk R, Najgebauer A, Pierzchała D, Jędrzejowicz WP, Nguyen NT, Hoang K (2011). Modelling and simulation of an infection disease in social networks. Computational collective intelligence technologies and applications.

[CR31] Khan T, Michalas A, Akhunzada A (2021). Fake news outbreak 2021: Can we stop the viral spread?. J Netw Comput Appl.

[CR32] Li Q, Zhang Q, Si L, Liu Y (2019). Rumor detection on social media: Datasets, methods and opportunities. Proceedings of the Second Workshop on Natural Language Processing for Internet Freedom Censorship, Disinformation, and Propaganda.

[CR33] Luo W, Tay WP (2012) Identifying multiple infection sources in a network. In: 2012 conference record of the forty sixth asilomar conference on signals, systems and computers (ASILOMAR), 1483–1489. 10.1109/ACSSC.2012.6489274.

[CR34] Luo W, Tay WP, Leng M (2014). How to identify an infection source with limited observations. IEEE Journal of Selected Topics in Signal Processing.

[CR35] Market chaos after fake Obama explosion tweet—ABC News (Australian Broadcasting Corporation). (b.d.). https://www.abc.net.au/news/2013-04-24/ap-twitter-feed-hacked/4647630?nw=0.

[CR36] Li M, Wang X, Gao K, Zhang S (2017). A survey on information diffusion in online social networks: Models and methods. Information.

[CR37] Newman MEJ, Girvan M (2004). Finding and evaluating community structure in networks. Phys Rev E.

[CR38] Nguyen HT, Ghosh P, Mayo ML, Dinh TN (2016) Multiple infection sources identification with provable guarantees. ArXiv:1608.06492* [Cs]*. http://arxiv.org/abs/1608.06492

[CR39] Peixoto TP (2014). Efficient Monte Carlo and greedy heuristic for the inference of stochastic block models. Phys Rev E.

[CR40] Pennycook G, Rand DG (2021). The psychology of fake news. Trends Cogn Sci.

[CR41] Pizzuti C, Rudolph WG, Jansen T, Beume N, Lucas S, Poloni C (2008). GA-Net: A Genetic algorithm for community detection in social networks. Parallel problem solving from nature—PPSN X.

[CR42] Pons P, Latapy M (2005) Computing communities in large networks using random walks. In: Pinar Yolum W, Güngör T, Gürgen F, Özturan C (eds) Computer and information sciences—ISCIS 2005, vol 3733. Springer, pp 284–293. 10.1007/11569596_31

[CR43] Prakash BA, Vreeken J, Faloutsos C (2012) Spotting culprits in epidemics: How many and which ones? In: 2012 IEEE 12th international conference on data mining, 11–20. 10.1109/ICDM.2012.136.

[CR44] Prat-Pérez A, Dominguez-Sal D, Larriba-Pey J-L (2014) High quality, scalable and parallel community detection for large real graphs. In: Proceedings of the 23rd international conference on world wide web - WWW ’14, 225–236. 10.1145/2566486.2568010.

[CR45] Raj PMK, Mohan A, Srinivasa KG (2018). Practical social network analysis with python.

[CR46] Reichardt J, Bornholdt S (2006). Statistical mechanics of community detection. Phys Rev E.

[CR47] Rossetti G, Milli L, Cazabet R (2019). CDLIB: A python library to extract, compare and evaluate communities from complex networks. Applied Network Science.

[CR48] Rosvall M, Bergstrom CT (2008). Maps of random walks on complex networks reveal community structure. Proc Natl Acad Sci.

[CR49] Rozemberczki B, Davies R, Sarkar R, Sutton C (2019) GEMSEC: graph embedding with self clustering. arXiv:1802.03997* [cs]*. http://arxiv.org/abs/1802.03997

[CR50] Ruan J, Zhang W (2007) An efficient spectral algorithm for network community discovery and its applications to biological and social networks. In: Seventh IEEE international conference on data mining (ICDM 2007) , 643–648. 10.1109/ICDM.2007.72.

[CR51] Ryan A Rossi, Nesreen KA (2015) The network data repository with interactive graph analytics and visualization. In: Proceedings of the twenty-ninth AAAI conference on artificial intelligence.

[CR52] Şen F, Wigand R, Agarwal N, Tokdemir S, Kasprzyk R (2016). Focal structures analysis: Identifying influential sets of individuals in a social network. Soc Netw Anal Min.

[CR53] Shah D, Zaman T (2010) Detecting sources of computer viruses in networks: Theory and experiment. In: Proceedings of the ACM SIGMETRICS international conference on measurement and modeling of computer systems—SIGMETRICS ’10 , 203. 10.1145/1811039.1811063.

[CR54] Shah D, Zaman T (2011). Rumors in a Network: Who’s the culprit?. IEEE Trans Inf Theory.

[CR55] Shelke S, Attar V (2019). Source detection of rumor in social network—A review. Online Social Networks and Media.

[CR56] Shu K, Mahudeswaran D, Wang S, Lee D, Liu H (2019) FakeNewsNet: a data repository with news content, social context and spatialtemporal information for studying fake news on social media. ArXiv:1809.01286* [Cs]*. http://arxiv.org/abs/1809.01286. 10.1089/big.2020.0062 32491943

[CR57] Stanford Large Network Dataset Collection. (b.d.). Pobrano 6 maj 2021, z http://snap.stanford.edu/data/.

[CR58] Tarapata Z, Kasprzyk R, Szczuka WM, Kryszkiewicz M, Ramanna S, Jensen R, Hu Q (2010). Graph-based optimization method for information diffusion and attack durability in networks. Rough sets and current trends in computing.

[CR59] Traag VA, Aldecoa R, Delvenne J-C (2015). Detecting communities using asymptotical surprise. Phys Rev E.

[CR60] Traag V, Waltman L, van Eck NJ (2019). From Louvain to Leiden: Guaranteeing well-connected communities. Sci Rep.

[CR61] Yang K, Shekhar AH, Oliver D, Shekhar S, Nascimento WMA, Sellis T, Cheng R, Sander J, Zheng Y, Kriegel H-P, Renz M, Sengstock C (2013). Capacity-constrained network-voronoi diagram: A summary of results. Advances in spatial and temporal databases.

[CR62] Zang W, Zhang P, Zhou C, Guo L (2014). Discovering multiple diffusion source nodes in social networks. Procedia Computer Science.

[CR63] Zang W, Zhang P, Zhou C, Guo L (2015). Locating multiple sources in social networks under the SIR model: A divide-and-conquer approach. Journal of Computational Science.

[CR64] Zhang P, Moore C (2014). Scalable detection of statistically significant communities and hierarchies, using message passing for modularity. Proc Natl Acad Sci.

[CR65] Zhang Z, Xu W, Wu W, Du D-Z (2017). A novel approach for detecting multiple rumor sources in networks with partial observations. J Comb Optim.

[CR66] Zhu K, Ying L (2013) Information source detection in the sir model: a sample path based approach. ArXiv:1206.5421* [Physics]*. http://arxiv.org/abs/1206.5421

